# Supplementary material for: Hypothalamus volumes in adolescent Myalgic Encephalomyelitis/Chronic Fatigue Syndrome (ME/CFS): impact of self-reported fatigue and illness duration
Source: Brain Struct Funct. 2023 Aug 3;228(7):1741–54. doi: 10.1007/s00429-023-02682-3 (PMC10471696; doi:10.1007/s00429-023-02682-3)
Supplement: Supplementary file 1 — Supplementary file1 (DOCX 13 KB) [file 429_2023_2682_MOESM1_ESM.docx]

**Supplementary Table 1.** Table adapted from Billot et al. (2020). Grouping of hypothalamic subunit nuclei, based on Bocchetta et al. (2015) and Makris et al. (2013).

| **Hypothalamus subunit** | **Subunit nuclei** |
| --- | --- |
| Anterior-superior | preoptic area; paraventricular nucleus |
| Anterior-inferior | suprachiasmatic nucleus; supraoptic nucleus |
| Superior tubular | dorsomedial nucleus; paraventricular nucleus; lateral hypothalamus |
| Inferior tubular | arcuate nucleus; ventromedial nucleus; supraoptic nucleus; lateral tubular nucleus; tuberomamillary nucleus |
| Posterior | mamillary body (including medial and lateral mamillary nuclei); lateral hypothalamus; tuberomamillary nucleus |
